# Shepherding the past: High-resolution data on Neolithic Southern Iberian livestock management at Cueva de El Toro (Antequera, Málaga)

**DOI:** 10.1371/journal.pone.0299786

**Published:** 2024-04-03

**Authors:** Alejandro Sierra, Vanessa Navarrete, Roger Alcàntara, María Dolores Camalich, Dimas Martín-Socas, Denis Fiorillo, Krista McGrath, Maria Saña

**Affiliations:** 1 Departament de Prehistòria, Laboratori d’Arqueozoologia, Universitat Autònoma de Barcelona, Cerdanyola del Vallès, Spain; 2 Institución Milá y Fontanals de Estudios en Humanidades del Consejo Superior de Investigaciones Científicas, Grupo de Arqueología de las Dinámicas Sociales, Barcelona, Spain; 3 School of History, Archaeology and Religion, Cardiff University, Cardiff, United Kingdom; 4 Departamento de Geografía e Historia, Área de Prehistoria, Universidad de la Laguna, Santa Cruz de Tenerife, Spain; 5 AASPE «Archéozoologie, Archéobotanique: Sociétés, Pratiques, Environnements» CNRS, MNHN, Paris, France; 6 Institute of Environmental Science and Technology (ICTA - UAB), Universitat Autònoma de Barcelona, Cerdanyola del Vallès, Spain; University of Padova: Universita degli Studi di Padova, ITALY

## Abstract

The feeding strategies of the first domesticated herds had to manage the risks arising from the novelty of livestock practices in territories often distant from the animals’ primary habitats. The Iberian Peninsula is characterised by a great diversity of environments, which undoubtedly influenced these dynamics. At the beginning of the Neolithic period these led the possibility to combine diverse livestock farming practices based on different animal feeding habits. This variability is also consistent with the rythms of adoption of domesticated animals, being later on the northern area. In order to address this issue, this work focuses on the dietary regimes of early sheep herds from southern Iberia, an area for which information is currently scarce. This study utilises high-resolution radiocarbon dating and stable isotope data on teeth to investigate sheep husbandry management strategies in Cueva de El Toro (Antequera, Málaga). The radiocarbon dates on the analysed remains evidenced they were deposited at the site over a short period, supporting the recurrent use of the cave. The sequential analysis of oxygen and carbon isotopes in tooth enamel reveals distinct livestock management strategies, reproduction patterns, feeding habits, and mobility during this short period. This variability demonstrates that livestock management practices in the western Mediterranean are more diverse than previously considered. Furthermore, these findings support the hypothesis that early Neolithic communities in the southern Iberian Peninsula were able to adopt different feeding strategies within the same herd, depending on their ecological and productive needs.

## Introduction

The spread of the farming economy across the Iberian Peninsula was a rapid process that took place between 5600 and 5400 cal BC [1–5]. The southeastern Iberian Peninsula followed this trend, with occupations being documented from the first half of the 6th millennium BC [6,7]. The socioeconomic data show diverse dynamics, highlighting the predominance of domestic caprines, mainly sheep, followed by pigs and cattle [8–11]. Most known sites are in caves [7] and they were likely used as stables. This is the case for Cueva de El Toro [12,13], and it has also been suggested for other sites such as Los Murciélagos de Zuheros [14]. This, together with functional studies [15,16], has led to suggest the existence of mobile pastoral communities [7,12] or communities with diverse degrees of mobility. It is therefore essential to study the feeding habits of domestic animals. However, the acquisition and procurement of feed for the animals cannot be understood in isolation from livestock management in general. Husbandry practices revolves around the control of three key aspects of animal biology: reproduction, feeding and mobility. Of these three aspects, feeding is fundamental because it ensures livestock production and herd survival. Farmers manage animal feeding behaviours for ecological, economic and social benefits [17].

Identifying the feeding regimes of early domestic herds in archaeology based on faunal remains is a complex issue. Livestock feeding can be organised in various ways depending on multiple factors, making it a multifaceted aspect with several interactions between communities, herds, and the environment [18,19]. Environmental diversity, the number of animals, access to pasture, and the degree of human dependence on livestock, as well as their relationship with agricultural practices, can condition herd feeding [20,21].

Changes in vegetation due to climate and seasonality affect the natural availability of pasture and nutrients, leading to adjustments throughout the annual cycle. Some of these adjustments may involve providing extra food or searching for new grazing areas [19]. In the first scenario, it is crucial to store fodder supplies, which necessitates coordinated planning with other agricultural tasks, occasionally involving the processing of fodder. The storage of fodder during periods of scarcity can undoubtedly be vital [22,23]. In mixed farming systems, stubble can serve as pasture [24,25]. In the second case, seasonal or altitudinal movements may be necessary to meet specific dietary needs that cannot be obtained in the vicinity of the settlement [26]. These movements can range from very limited to annual recurrences [27–30]. It is important to consider the conditions under which these movements occur, including their duration, seasonality, and motivation [31]. In archaeology, research on mobility strategies in livestock management has mainly focused on transhumance. However, it is important to note that transhumance is just one type of pastoral mobility.

The difficulty in detecting herd movements archaeologically is probably due to the lack of sufficient temporal resolution. Pastoral mobility, which refers to the movement of people and their herds, is an adaptive or reactive strategy to environmental variability in its broadest sense [19,32,33] and can take on a wide range of manifestations [27,30].

Seasonal movements between grazing areas to take advantage of seasonal pasture availability differ from extensive grazing. In extensive grazing, herds have access to large areas of natural pasture in the surrounding settlement landscape or within a more limited territorial extent. Different domestic animal species can share grazing areas, resulting in more efficient resource utilization [34,35]. The cyclical pattern of grazing areas would facilitate vegetation recovery in previously grazed areas [36,37]. In addition to pasture accessibility, other variables such as pasture quality and nutrient richness may directly influence livestock health and productivity [38]. The size of the herd, its diversity and the pressures on grazing land also influenced the feeding regimes implemented [37,39]. One of the main objectives is to prevent overexploitation of certain areas.

Livestock feeding strategies may also be influenced by factors such as predators, diseases, and conflicts with other communities. Livestock mobility may have been a response to these risks. Currently, there is controversy surrounding the differentiation of people and animals moving together and animal exchange practices [40]. Exchange was common in hunter-gatherer societies in the Iberian Peninsula [41] and could continue throughout the Neolithic period [42]. Archaeology has traditionally focused on the exchange of material culture, such as raw materials [43,44] and objects of adornment/prestige [45–47]. However, the exchange of live animals has received little attention, partly due to its limited archaeological visibility. The introduction of domestic species into certain regions may have been facilitated through exchange, as observed in ethnographic studies [48].

Neolithic communities adjusted their animal feeding strategies to cope with changing environmental and social challenges. It is crucial to maintain adaptive capacity for successful pastoral life [18]. Ethnographic studies have shown that animal feeding planning is determined by ecological, economic, political, social, and ideological factors [40,49]. Ben Hounet [50] states that pastoral systems are recognised for their capacity to adapt to evolving and demanding geo-political, economic, and social contexts. In prehistoric archaeology, animal mobility is often linked to environmental and climatic changes, such as ecological degradation [51], water shortages, erosion, or other climatic impacts on the territory. This suggests that the movement of herds and the search for new grazing areas may have played a role in adapting to changing environments. The various methods of organising pastoral ecology may have been used to manage perceived risks or uncertainties, reduce herd losses, ensure animal reproduction [52], and prevent disease and conflicts with other communities.

The development of stable isotope analysis on domestic animal remains [53] has addressed many of the issues raised so far. Recent studies suggest different livestock management strategies during the Early Neolithic. Examples of year-round use of pastures close to settlements include La Draga [54] and Chaves [55]. Vertical mobility of herds has been documented since the Neolithic, for example at sites such as Arene Candide in Italy [56] or Trocs in the Iberian Pyrenees [57]. Recently, a similar analysis was conducted on the faunal remains of the main domestic species at the Cueva de El Toro site. The analysis revealed a wide variability of δ^15^N and δ^13^C values in sheep bone collagen [11]. The study’s findings suggest several hypotheses regarding the feeding strategies of sheep flocks. These include mobility between non-cave grazing areas, access to crop fields, or even feeding on agricultural by-products in the stabling area during the cave occupation [11]. However, the temporal resolution in bulk collagen analysis is limited, making it unclear how much annual or seasonal variability exists in these feeding regimes. To gain a better understanding on this issue, it is important to conduct sequential carbon and oxygen isotope analysis of tooth enamel supported with carbon dating of the remains for higher temporal resolution.

Carbon and oxygen isotopic values are fixed in the teeth at the time of their formation, reflecting changes on a seasonal scale. The δ^18^O values are derived from water intake and plant consumption [58,59], and are directly related to temperature. In mid-latitudes, δ^18^O values are indicative of the warmer season if they are higher and of the cooler season if they are lower [60]. This seasonal variation allows estimation of the time of birth of the animals [61]. The δ^13^C values are derived from the diet of the plants consumed [62,63], and can distinguish between diets with plants with different photosynthetic pathways. In addition, plant values may vary depending on aspects such as water availability or altitude. The combination of δ^18^O and δ^13^C values allows to infer changes in diet within the annual cycle. Animals grazing in the same area year-round are expected to have covariant sequences [64]. However, deviations related to forage use or mobility can be observed [65]. One of these changes may be due to vertical mobility as animals grazing at higher altitudes in summer have lower δ^13^C sequences with higher δ^18^O values [66]. This results in a negative covariation of δ^18^O and δ^13^C sequences [57,67–69].

This work seeks to deepen our knowledge of livestock farming practices in the Early Neolithic, emphasising the variety of feeding strategies and reproductive management of herds. For this purpose, a sequential analysis of stable carbon and oxygen isotopes has been carried out on Cueva de El Toro sheep teeth. In addition, all the samples analysed were radiocarbon-dated. This approach contributes to a high-resolution temporal reconstruction of herds management practices. The combination of chronological and animal management data has contributed to unravelling the livestock management practices at the beginning of the Neolithic in the south of the Iberian Peninsula, providing new data for the whole western Mediterranean.

## Materials

**Fig 1:**
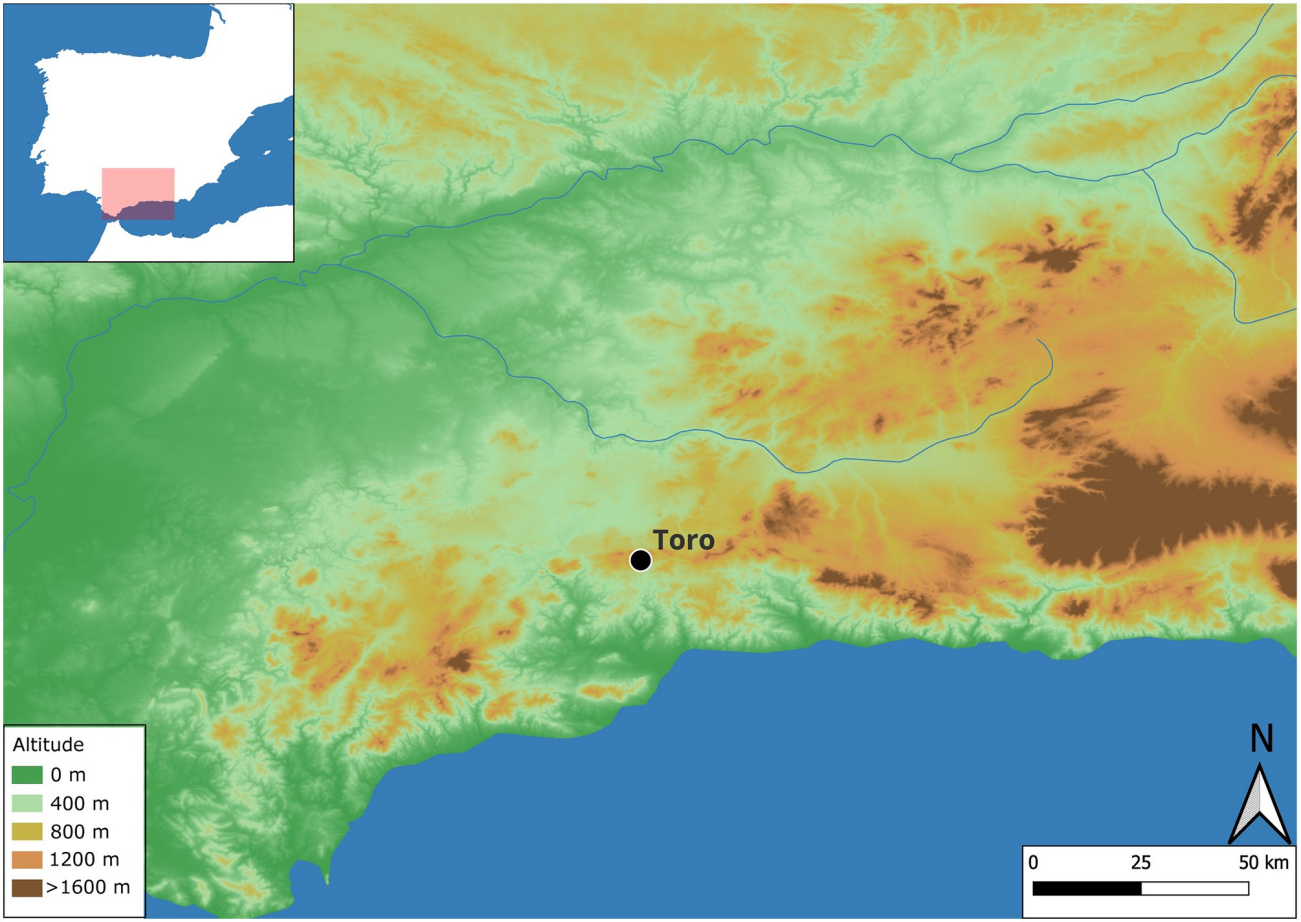
Location of Cueva de El Toro (Antequera, Málaga).

Cueva de El Toro (Antequera, Málaga) is a cave site at 1,190 masl and part of the wide karstic mountain range of calcareous rocks and diaclastic systems known as Sierra del Torcal (Fig 1). The cave is divided into two distinct areas: Sector 1 (upper) and Sector 2 (lower). In Sector 2, the stratigraphic sequence reveals four chrono-cultural phases, with the earliest two corresponding to Neolithic occupations [12]: Phase III (Late Neolithic, 4250–3950 2σ cal BC), subdivided into Phases IIIA and IIIB, and Phase IV (Early Neolithic, 5280–4780 2σ cal BC). The earliest occupation (Phase IV) suggests a farming context characterised by domestic animals, mainly caprines [11], and cereal plants [70], although gathering and hunting still played an important role. This phase has been interpreted as a seasonal occupation where husbandry practices had a dominant role. Micromorphological analysis revealed that the cave was synchronically used as a domestic space and to stable animals [12,13], with domestic activities related to processing animal carcasses and the preservation (smoking) of meat [12,15].

This work focuses on eight lower third molars (M_3_) selected from 24 to 72-month-old sheep (*Ovis aries*) individuals from Phase IV (5280–4780 2σ cal BC). Species attribution was confirmed morphologically, and by ZooMS analysis (Table 1). All eight samples were radiocarbon-dated to establish their chronology and identify the episode duration during which they were deposited. All necessary permits were obtained for the described study, which complied with all relevant regulations. The museum of Malaga and the Junta de Andalucia gave us the necessary permissions to carry out this work.

**Table 1:** Samples selected for isotope analysis.

ID	Phase	Species ZooMS	Teeth	Side	Payne (1973) age classes	Payne (1973) age
CTC1	IV	*Ovis aries*	M_3_	R	G	48–72 m
CTC2	IV	*Ovis aries*	M_3_	R	F	36–48 m
CTC4	IV	*Ovis aries*	M_3_	L	F	36–48 m
CTC5	IV	*Ovis aries*	M_3_	R	E	24–36 m
CTC7	IV	*Ovis aries*	M_3_	R	G	48–72 m
CTC8	IV	*Ovis aries*	M_3_	L	G	48–72 m
CTC19	IV	*Ovis aries*	M_3_	L	F	36–48 m
CTC21	IV	*Ovis aries*	M_3_	L	G	48–72 m

L: Left, R: Right; m = month.

## Methods

### Radiocarbon analysis

The sheep samples analysed were radiocarbon-dated at the CIRAM laboratory (France). The protocol used by the laboratory is described in S1 File. Bayesian chronological modelling of this radiocarbon dataset was performed using OxCal v.4.4 software [71]. Data was calibrated using the IntCal20 calibration curve [72]. As they belonged to the same phase, the model was defined by grouping all samples with start and end boundaries. The duration of the phases was calculated using the duration function. The OxCal model code is given in S1 Code. The Bayesian model was run five times and the results were compared to assess reproducibility. Estimates of the start, end and duration of each phase, plus the dates of each object within the stratigraphic units, are given in S1 Table.

### ZooMS

Zooarcheology by Mass Spectrometry (ZooMS), a method of peptide mass fingerprinting of collagen type I that is commonly used in archaeological analyses to provide taxonomic identifications [73], was performed on the eight lower molars to confirm the morphological identifications of sheep. For each molar, a small sample (between 10–30 mg) was taken and demineralized in 250 μl of 0.6M hydrochloric acid (HCl). Once demineralized, the acid was discarded and the samples were washed three times with 200 μl of 50 mM ammonium bicarbonate ((NH_4_)HCO_3_, AmBic, pH 8.0), and gelatinized in 100 μl of AmBic at 65°C for one hour. 50 μl was transferred to a new tube and the collagen was digested with 0.4 μg/μl of trypsin at 37°C for 18 hours. 1 μl of 5% trifluoroacetic acid (TFA) was added to stop the trypsin activity and the samples were desalted using C18 zip tips (100 μl, Pierce™ ThermoScientifc). 1 μl of sample was mixed with 1 μl of matrix solution (α-cyano-4-hydroxycinnamic acid) and spotted in triplicate on a AB 384 Opti-TOF steel target plate then analyzed on an AB SCIEX 4800 MALDI-ToF-MS. Spectra were averaged and analyzed using mMass [74], and identified using known references [73,75–77].

### Carbon and oxygen stable isotopes analysis

Stable oxygen (δ^18^O) and carbon (δ^13^C) isotope analysis of tooth enamel bioapatite was carried out to investigate sheep management patterns of Cueva de El Toro. The sampling method followed the procedure outlined in [78] and involved sequential sampling of the buccal side middle lobe of M_3_. To access the enamel surface, a tungsten drill was used to abrade it gently. Sequential enamel samples were taken at 1–1.5 mm intervals from the apex to the enamel-root junction in a perpendicular direction to the tooth growth axis. A low magnification lens (×3) was used to ensure accuracy. The location of the samples within the tooth crown was determined by measuring their distance from the enamel-root junction.

Enamel carbonate samples were pre-treated, following the protocol described in Balasse et al. [79] and modified by Tornero et al. [80], for 4 hours using 0.1 M acetic acid (CH_3_COOH) at a ratio of 100 μl of solution per 100 μg of sample, resulting in a 34.7 ± 5% weight loss. Samples of pre-treated enamel powders of around ~600 μg were reacted with 100% phosphoric acid at 72 °C for 360 seconds in individual vessels in an automated cryogenic distillation system (Kiel IV device) interfaced with a DeltaVAdvantage isotope ratio mass spectrometer. The precision and accuracy of the measurements were verified with the calcium carbonate standard of the laboratory (Marbre LM standardised to the NBS 19 international standard). Stable isotopes were analysed at the SSMIM in Paris. The results are expressed in V-PDB, with an estimated analytical precision of 0.03‰ for δ^18^O values and 0.02‰ for δ^13^C values (the mean of the standard deviations of all samples analyzed), based on four to eight Marbre LM analyses for each run. Throughout the analysis, 66 Marbre LM samples gave an average δ^18^O value of -2.07 ± 0.05‰ and an average δ^13^C value of 2.18 ± 0.03‰.

The δ^18^O sequences were modelled to remove inter-individual variability in tooth size, using a four-parameter equation derived from a cosine function, as described in Balasse et al. [61]. The four parameters used were the position of the maximum value of δ^18^O (x0), the cycle period (X), the signal amplitude (A), and the mean (M). X is the period (in mm), corresponding to the length of the tooth crown potentially formed during an entire annual cycle; x0 is the position of δ^18^Omax at the tooth crown indicating the delay. The period (X) is used to normalise the position (x0) of δ^18^Omax at the crown of the tooth. A is the amplitude [= (max.—min.)/2] (in ‰) and M is the mean [= (max. + min.)/2] expressed in ‰. The inter-individual variability in the ratio (x0/X) refers to the distribution of births, which are compared to pre-existing reference sets for the third molar [59,60–62].

## Results

### Bayesian chronological modelling

**Fig 2:**
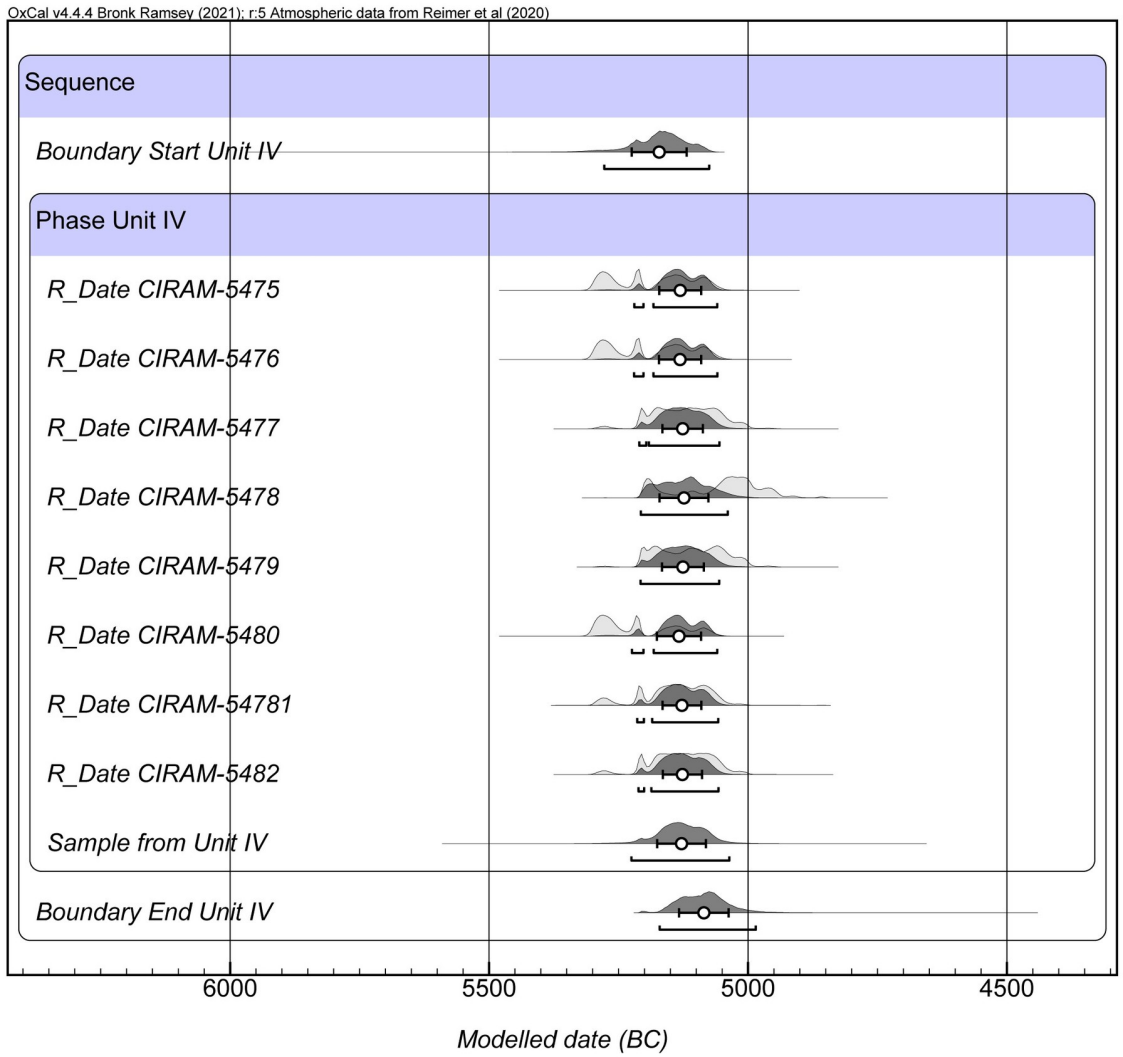
Bayesian chronological model for the accumulation of analysed sheep.

All eight samples produced reliable dates associated with Phase IV of Cueva de El Toro. All dates sit within the second half of the 6th-millennium cal BC (Table 2 and Fig 2). The duration of the accumulation event for all individuals was ascertained using Bayesian modelling. The model performed with all eight datations, marked sample CIRAM-5478 as an outlier. The model was Re-ran excluding this sample, and similar results were obtained (S1 Table). Both Bayesian models at 95.4% confidence provided a high chronological resolution, evidencing that the individuals were deposited within 239.2 ± 2.5 years with all dates or 239 ± 8.4 years without the outlier (Fig 2 and S1 Table).

**Table 2:** Radiocarbon dates of Cueva de El Toro.

Lab code	Sample	Conventional age BP	±	cal BC 2σ (95%)
CIRAM-5475	CTC1	6230	35	5306–5056
CIRAM-5476	CTC2	6232	35	5306–5058
CIRAM-5477	CTC4	6172	35	5215–5009
CIRAM-5478	CTC5	6113	36	5209–4939
CIRAM-5479	CTC7	6158	33	5211–5006
CIRAM-5480	CTC8	6242	33	5307–5068
CIRAM-5481	CTC19	6201	33	5296–5043
CIRAM-5482	CTC21	6185	33	5285–5016

### ZooMS

ZooMS confirmed the morphological identifications of sheep for all eight molars. Due to poor spectral resolution at a key peptide marker for distinguishing between sheep and certain deer species (marker ɑ2 502, with *m/z* 1580 for sheep and *m/z* 1550 for deer), several samples were given an identification of “sheep/deer”, with “deer” including red deer (*Cervus elaphus*), fallow deer (*Dama dama*) or European elk (*Alces alces*). As the remains had been morphologically classified as *Ovis/Capra*, all samples were finally identified as sheep. Peptide markers used to make the identifications for all samples can be found in S6.

### Carbon and oxygen isotope ratios

**Fig 3:**
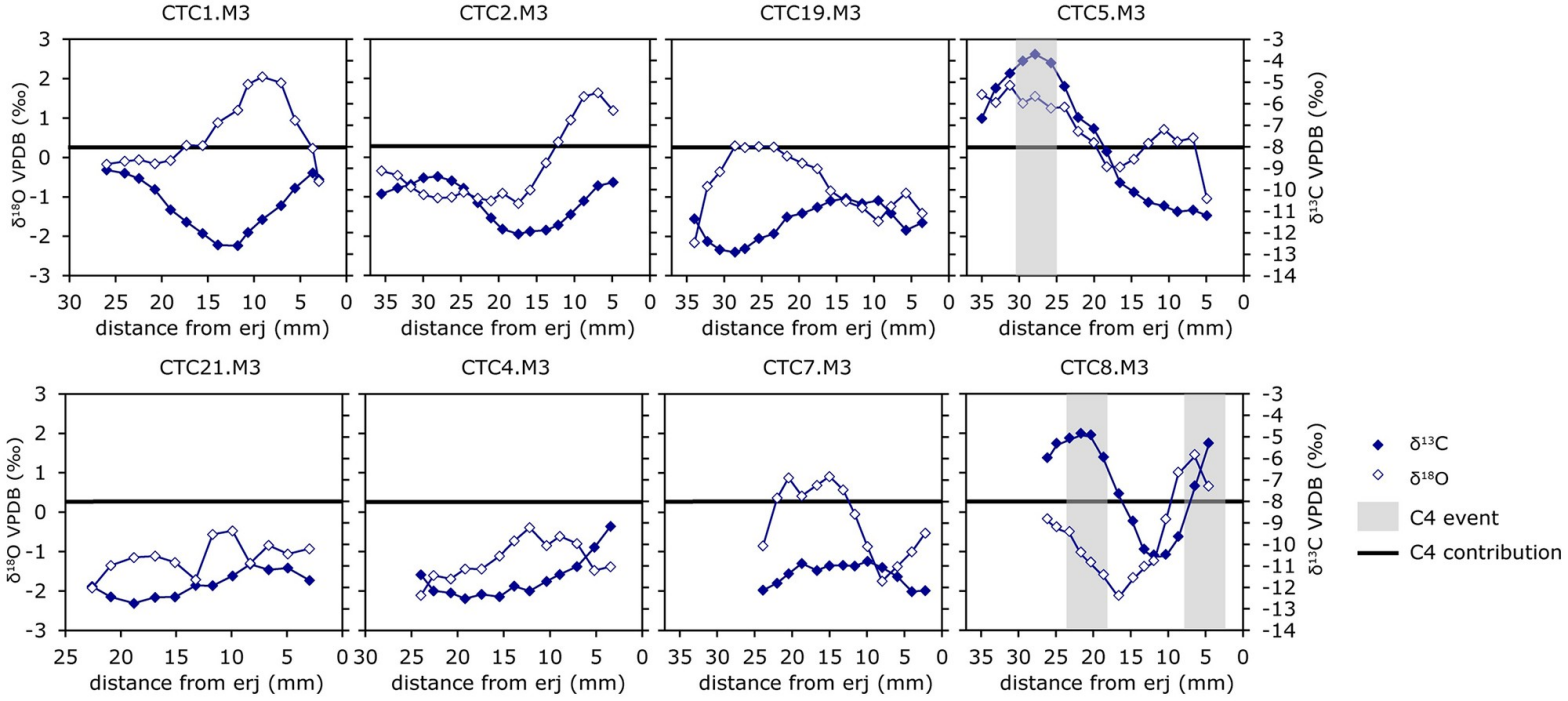
Sequences of the values δ^18^O and δ^13^C measured in the tooth enamel of M_3_ from Cueva de El Toro’s sheep.

Stable carbon and oxygen isotope ratios are shown in Fig 3 and S2 Table. Overall, the δ^18^O values vary between -2.16‰ and 2.05‰, the mid-range (M) value varies between 1.25‰ and 0.72‰, and the amplitude of intra-tooth variation is between 1.45‰ and 3.58‰ (Table 3). The δ^13^C values vary between -12.91‰ and -3.69‰, the mid-range value varies between -11.83‰ and -7.44‰, and the intra-tooth variation is between 1.41‰ and 7.51‰ (Table 3). Individuals CTC5 and CTC8 have very high carbon values during part of the sequence. Most of the individuals have very different δ^18^O and δ^13^C sequences, as can be seen in Fig 3. One-way ANOVA revealed statistically significant differences between individuals for both δ^18^O (F(7, 111) = 10.62, p < 0.05) and δ^13^C (F(7, 111) = 20.74, p < 0.05). In addition, Tukey’s test revealed statistically significant differences between individuals (S3 and S4 Tables).

**Table 3:** Tooth enamel carbon and oxygen stable isotope ratios from Cueva del Toro M = (Min+Max)/2.

	δ^18^O_VPDB COR_				δ^13^C_VPDB COR_			
	Min	Max	M	A	Min	Max	M	A
CTC1	-0.61	2.04	0.72	2.65	-12.60	-9.08	-10.84	3.52
CTC2	-1.17	1.64	0.23	2.81	-12.07	-9.39	-10.73	2.68
CTC19	-2.16	0.30	-0.93	2.46	-12.91	-10.40	-11.66	2.51
CTC5	-1.05	1.83	0.39	2.88	-11.20	-3.69	-7.44	7.51
CTC21	-1.92	-0.47	-1.20	1.45	-12.74	-10.92	-11.83	1.82
CTC4	-2.11	-0.39	-1.25	1.72	-12.52	-9.16	-10.84	3.36
CTC7	-1.75	0.91	-0.42	2.65	-12.20	-10.78	-11.49	1.41
CTC8	-2.11	1.47	-0.32	3.58	-10.48	-4.82	-7.65	5.66

The δ^18^O and δ^13^C sequences of all the individuals compared using Pearson’s r correlation test show a great diversity in δ^18^O- δ^13^C correlation (Table 4). Only in 3 individuals do both sequences correlate with each other. Individual CTC5 is positively correlated, while CTC1 and CTC19 are negatively correlated.

**Table 4:** Statistical data of the δ^18^O and δ^13^C cycle. Correlation coefficient (Pearson’s r) and significance level (p). The significant results are in bold.

Pearson	r	p
CTC1	-0,58	**0,02**
CTC2	0,09	0,72
CTC19	-0,57	**0,02**
CTC5	0,78	**0**
CTC21	0,24	0,44
CTC4	-0,08	0,8
CTC7	0,3	0,32
CTC8	0,04	0,89

In the rest of the individuals, δ^18^O and δ^13^C sequences do not correlate.

The intra-tooth δ^18^O sequences for M3 vary according to a sinusoidal pattern, probably reflecting the seasonal cycle. The lowest values indicate the winter, and the highest mark the summer.

### Modelling of the δ^18^O sequences

**Fig 4:**
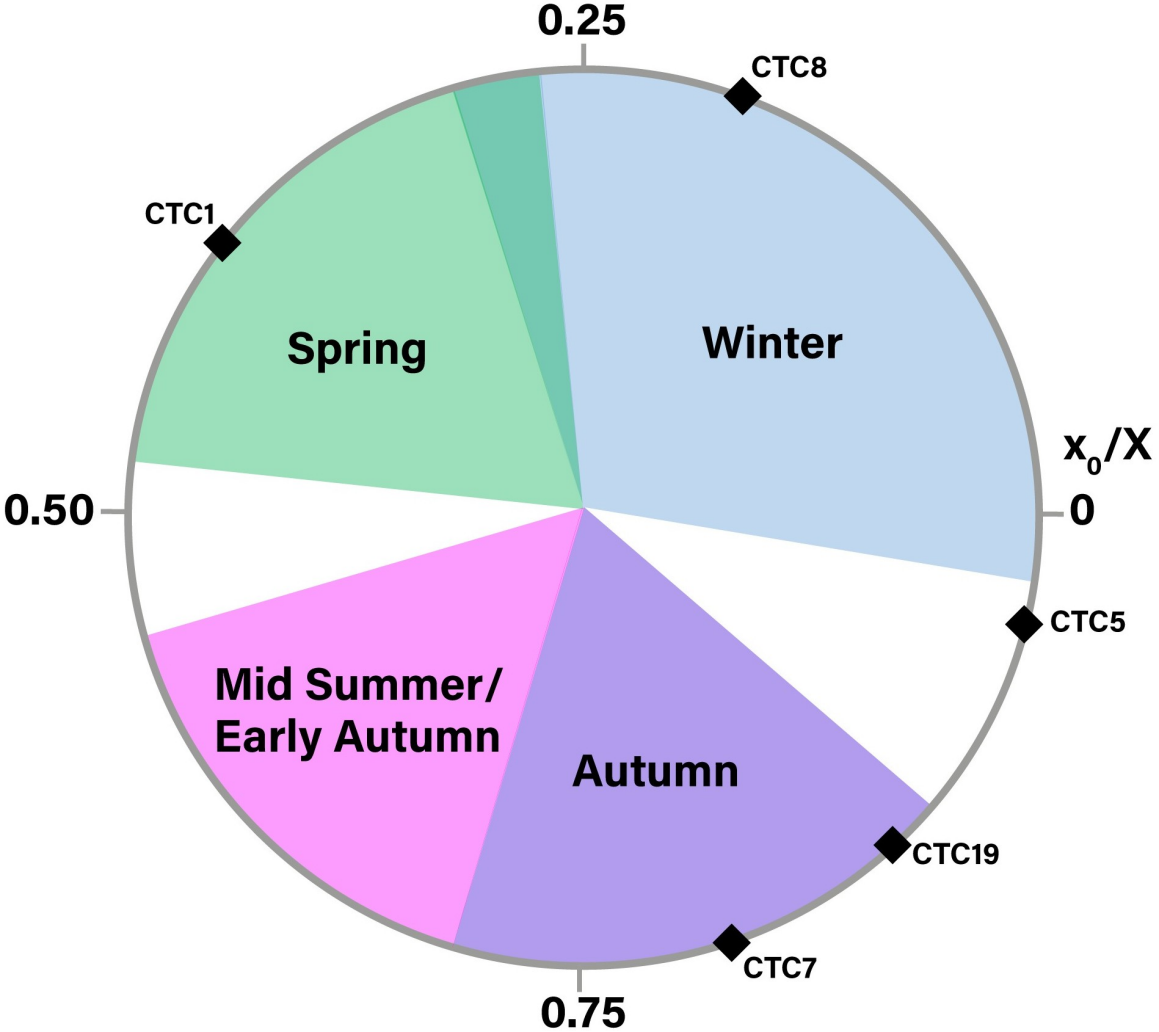
Distribution of sheep births at Cueva de El Toro, as reflected by the position of the maximum δ^18^O value in tooth crown (x0) normalised to the period of the cycle (X). Season of birth is compared to modern reference sheep (Carmejane: [81]; Rousay: [82,83]; Le Merle and La Fage: [84]). Blue, green, purple and pink color areas represent x0/X ratios obtained from modern specimens.

Results from modelling the δ^18^O sequences and the normalised location of the δ^18^O sequence optimum (x0/X) in the tooth crown are summarised in S5 Table. Only four individuals could be modelled (CTC1, CTC19, CTC5 and CTC7). The other individuals could not be modelled due to truncated sequences. The individuals that could be modelled show values corresponding to very different times of the year when compared to current known birth date references [81–84] (Fig 4). Two individuals (CTC19 and CTC7) show values of 0.80 and 0.90, corresponding to autumn births. On the other hand, two other individuals (CTC1 and CTC5) have values of 0.02 and 0.49, corresponding to winter and spring births, respectively.

## Discussion

The combination of radiocarbon data and carbon and oxygen stable isotope values on sheep teeth has framed high-resolution information on animal management practices over an archaeological high temporal resolution. The radiocarbon dating of the teeth of the eight sheep individuals analysed and its Bayesian analysis show that all the individuals were killed within a period of 240 years. Regarding the isotopic values, the δ^18^O sequences present high inter-individual variability. This large diversity may be due to different breeding patterns and feeding strategies.

The values of δ^13^C results also show highly variable sequences. Two individuals (CTC5 and CTC8) are documented to have diets that may have a significant contribution of C_4_ plants (>-8‰, [85]), at least during the summer. C_4_ plants are well adapted to arid conditions, and are present in small percentages is some areas of southern Europe, such as the Iberian Peninsula [86,87]. These plants are present in the area’s vegetation today [88]. Pollen studies for the southern Iberian Peninsula also find them in small percentages throughout the Holocene [89]. Some authors have documented this in the isotopic analyses of Neolithic sheep from Cueva de Nerja [90]. In addition, a series of salt ponds are located in the area surrounding Cueva de El Toro [91,92]. Such formations are host to halophytic plants with C_4_ photosynthetic pathways [93]. Several possibilities may explain the C_4_ values obtained. The sheep may have grazed in areas with C_4_ plants of the *Chenopodiaceae* family. However, even if they have not been found at the site [70] it cannot be ruled out that these plants were used as fodder in summer. Species such as the Mediterranean saltbush (*Atriplex halimus*) are essential from a foraging point of view [94]. This fodder may have been a feed supplement in the context of a stable [13]. Another possibility is that sheep grazed during summer in the salt marshes, with the C_4_ plants, found in the region. They could have also grazed near the sea [95], which is 35 km away and is known to have been visited by human groups [96]. However, the existence of C_4_ environments near the site provides a more solid argument. In addition, we must bear in mind that even the highest δ^13^C values correspond to a diet comprising less than 50% of C_4_ plants [97]. In any case, this type of diet only seems to be observed in two of the individuals studied (CTC5 and CTC8) during part of the year. The rest of the individuals present carbon values that support the consumption of C_3_ plants throughout the year. However, the variation in δ^13^C sequences is very different between individuals, likely resulting from different feeding regimes throughout the annual cycle.

The δ^18^O and δ^13^C sequences of individuals CTC1 and CTC19 are negatively correlated. The negative correlation has been previously interpreted in the Iberian Peninsula and Italy as reflecting vertical mobility towards mountain pastures due to depleting δ^13^C values along an altitudinal gradient [56,57,66,98].

In summary, all individuals show very different δ^18^O and δ^13^C sequences. Most of the δ^18^O sequences are very different between individuals. The same applies to the δ^13^C sequences. Furthermore, the results of Pearson’s correlation test shows that five of the individuals have uncorrelated δ^18^O and δ^13^C sequences, while one has a positive correlation (CTC5) and two others have a negative correlation (CTC1 and CTC19). These differences between individuals have also been observed through bulk collagen stable isotopes analysis [11]. In addition, the season of birth modelling, as well as its comparison with current published references [81–84], shows diverse reproductive patterns, with individuals being born in autumn, winter and spring. This reveals that human groups controlled the reproduction of their animals so that births took place when it was most convenient for human interests [99]. These data reinforce the hypothesis based on the presence of young individuals that the cave was used as a breeding ground [11].

Therefore, great diversity in terms of reproduction, feeding and mobility can be observed among sheep individuals. These results lead us to consider what herd management strategies occurred at Cueva de El Toro. The available data supports that each sheep was likely herded differently. This contrasts with what has been observed at sites such as La Draga [54] or Chaves [55], where little variability in isotope values is observed. This contrasting difference may be due to feeding strategies being implemented to adapt to different access to resources and the varying socio-economic strategies dominating at each site. At the same time, the multiple patterns observed in the sheep from Cueva de El Toro reflect different herding strategies. These strategies may include altitudinal mobility, as suggested by the negative covariance in two individuals. The existence of pastoral mobility in Cueva de El Toro has previously been proposed. The archaeological evidence shows a low-intensity occupation during phase IV and supports its use as a sheep and goat corral [13]. In addition, the data show how the lithic industry arrived at the site as a finished product [15]. The presence of marine molluscs consumed and used for pottery or ornaments also reinforce the hypothesis of mobile groups [96].

Risk management strategies are another element to consider when talking about management strategies. Risk management strategies enable coping with constraints related to the environment or the biological cycles, but also with other situations such as epidemics or social conflicts (e.g. livestock theft, grazing conflicts, etc.) [100]. One of these risk management mechanisms is livestock diversification [101]. This diversification can take many forms: e.g. multiple periods of birth or different feeding regimes, the division of the herd into smaller groups to exploit different areas or the use of seasonal feed supplements [37]. In any case, the isotopic evidence from Cueva de El Toro shows the existence of some of these practices at the site diversification of birth season, different feeding patterns, and herd movement.

However, these detected practices can also be explained as an adaptive response of early farming societies. Adaptation has been linked to changes in climatic conditions, but may also occur with other socio-economic, political and cultural transformations [102]. In any case, the adaptability of livestock systems rests on two fundamental pillars: breeding organisation and feed diversification. Flexibility within these two pillars allows farmers to modify herd management each year, thus reducing uncertainty and ensuring the sustainable use of available resources [103]. The diversity observed in dietary regimes could be explained by livestock accessing nearby pastures with different δ^13^C values due to changes in environmental conditions. From the second half of the 6th millennium BC, the landscape of Sierra de El Torcal changed with the progressive anthropisation [104].

In summary, the explanations for the wide variability of herding strategies can be very diverse and not necessarily disconnected: livestock production strategies, risk management strategies or adaptation to changing constraining factors. All these explanations are feasible with the data currently available. The results show a greater diversity of management strategies in the Early Neolithic than previously thought [54,55,105]. The archaeozoological and collagen δ^13^C and δ^15^N isotopic study of Cueva de El Toro [11] already pointed out that the different domestic species were managed differently and that the variability of sheep values also indicated different management strategies. New results from the δ^13^C and δ^18^O sequence analysis of sheep teeth support this diversity in management strategies, as has been observed at other Neolithic sites [65]. In this case, some of these strategies may involve mobility, as previously suggested from other archaeological data [13,15,96]. In this sense, Cueva de El Toro would have played the role of a sheepfold cave in which to shelter livestock, as shown by the presence of abundant young caprines [11] and micromorphology studies [13]. Nevertheless, it appears that the cave’s use was more intricate than a mere enclosure for caprines. The abundance of pig remains and the available isotopic data indicate that pigs were likely raised extensively in different grazing areas near the cave, consuming animal protein as part of their diet [11].

The diverse herding strategies documented at Cueva de El Toro fit well within the economic model proposed for the Early Neolithic communities of the southern Iberian Peninsula, which have been considered highly mobile pastoralist communities [7,12]. Despite the scarcity of isotopic data, the abundance of caprines can support this hypothesis. In addition, the available data on pottery production and provisioning [46,106], lithic tools [107] and ornamental raw materials [108–111] also seem to align with this proposal. This work supports this hypothesis, showing a wide diversity of livestock management modes, including altitudinal mobility practices, at the same site and over a short period of time.

## Conclusion

Integrating radiocarbon, archaeozoology and dental enamel stable isotope data provided high-resolution information to study animal husbandry strategies. Radiocarbon results on eight sheep individuals from Cueva de El Toro, identified by archaeozoology and ZooMS, show how they were deposited at the site over a short period. Tooth enamel isotope results support different livestock management strategies with different breeding, feeding and mobility patterns occurring within this archaeological short period. This variability may have resulted from many factors. Still, it shows how livestock management practices in the western Mediterranean were much more diverse than previously thought and reinforces the hypothesis that the early Neolithic communities of southern Iberia were characterised by their complexity. This study enabled the characterisation of the animal husbandry practices of the first farming groups in Cueva de El Toro through stable isotopes in the enamel, helping to unravel what were animal husbandry practices like in southern Iberia.

## Supporting information

S1 Table Results of the Bayesian model. (XLSX)

S2 Table Individual isotope results for each sampled specimen. (XLSX)

S3 Table Statistical tests of δ^18^O stable isotope results. Significant differences in bold.
(DOCX)

S4 Table Statistical tests of δ^13^C stable isotope results. Significant differences in bold.
(DOCX)

S5 Table Modelling of δ^18^O sequences. Results from the calculation of the best fit for combined variation of X (period), A (amplitude), x0 (delay), M (mean) and Pearson correlation.
(DOCX)

S1 Code Bayesian model code for the radiocarbon dates of the Cueva de El Toro. (DOCX)

S1 File Protocol for carrying out radiocarbon analysis and results of each sample with the quality control data. (DOCX)
