# Supplementary material for: Shepherding the past: High-resolution data on Neolithic Southern Iberian livestock management at Cueva de El Toro (Antequera, Málaga)
Source: PLoS One. 2024 Apr 3;19(4):e0299786. doi: 10.1371/journal.pone.0299786 (PMC10990244; doi:10.1371/journal.pone.0299786)
Supplement: S3 Table — Significant differences in bold. (DOCX) [file pone.0299786.s003.docx]

**S3 Table. Statistical tests of δ^18^O stable isotope results.** One-way ANOVA and Tukey test. Significant differences in bold

| One-way ANOVA | |  |  |  |  |
| --- | --- | --- | --- | --- | --- |
|  |  |  |  |  |  |
|  | Sum of sqrs | df | Mean square | F | p (same) |
| Between groups: | 50.383 | 7 | 7.19758 | 10.62 | **3.61E-10** |
| Within groups: | 75.2631 | 111 | 0.678046 | Permutation p (n=99999) | |
| Total: | 125.646 | 118 | 1.00E-05 |  |  |

| Tukey test | CTC1 | CTC2 | CTC19 | CTC5 | CTC21 | CTC4 | CTC7 | CTC8 |
| --- | --- | --- | --- | --- | --- | --- | --- | --- |
| CTC1 |  | 0.07841 | **0.001617** | 0.9999 | **1.29E-05** | **3.14E-06** | 0.2211 | **0.006044** |
| CTC2 | 4.129 |  | 0.8844 | **0.01655** | 0.09989 | **0.04865** | 1 | 0.9605 |
| CTC19 | 5.874 | 1.884 |  | **0.0001734** | 0.7525 | 0.5953 | 0.8281 | 1 |
| CTC5 | 0.6072 | 4.904 | 6.693 |  | **1.10E-06** | **2.22E-07** | 0.07266 | **0.0008967** |
| CTC21 | 7.568 | 3.992 | 2.255 | 8.344 |  | 1 | 0.09501 | 0.6879 |
| CTC4 | 8.02 | 4.383 | 2.6 | 8.832 | 0.2694 |  | **0.04913** | 0.5313 |
| CTC7 | 3.488 | 0.3348 | 2.061 | 4.171 | 4.02 | 4.378 |  | 0.9245 |
| CTC8 | 5.344 | 1.522 | 0.2632 | 6.098 | 2.403 | 2.734 | 1.725 |  |
