# Supplementary material for: Shepherding the past: High-resolution data on Neolithic Southern Iberian livestock management at Cueva de El Toro (Antequera, Málaga)
Source: PLoS One. 2024 Apr 3;19(4):e0299786. doi: 10.1371/journal.pone.0299786 (PMC10990244; doi:10.1371/journal.pone.0299786)
Supplement: S4 Table — Significant differences in bold. (DOCX) [file pone.0299786.s004.docx]

**S4 Table. Statistical tests of δ^13^C stable isotope results.** One-way ANOVA and Tukey test. Significant differences in bold

| One-way ANOVA | |  |  |  |  |
| --- | --- | --- | --- | --- | --- |
|  |  |  |  |  |  |
|  | Sum of sqrs | df | Mean square | F | p (same) |
| Between groups: | 344,542 | 7 | 49,2202 | 20,74 | **1,30E-17** |
| Within groups: | 263,482 | 111 | 2,37371 | Permutation p (n=99999) | |
| Total: | 608,023 | 118 | 1,00E-05 |  |  |

|  | CTC1 | CTC2 | CTC19 | CTC5 | CTC21 | CTC4 | CTC7 | CTC8 |
| --- | --- | --- | --- | --- | --- | --- | --- | --- |
| CTC1 |  | 1 | 0.7565 | **4.76E-06** | 0.5146 | 0.7763 | 0.9272 | **8.69E-07** |
| CTC2 | 0.218 |  | 0.6062 | **2.97E-06** | 0.3721 | 0.6443 | 0.8515 | **5.38E-07** |
| CTC19 | 2.246 | 2.578 |  | **7.77E-10** | 0.9995 | 1 | 1 | **1.82E-10** |
| CTC5 | 7.888 | 8.037 | 10.47 |  | **1.64E-09** | **6.97E-09** | **3.91E-08** | 0.9979 |
| CTC21 | 2.769 | 3.082 | 0.7346 | 10.26 |  | 0.9999 | 0.9952 | **3.65E-10** |
| CTC4 | 2.197 | 2.497 | 0.1007 | 9.844 | 0.5993 |  | 1 | **1.49E-09** |
| CTC7 | 1.712 | 1.992 | 0.3982 | 9.345 | 1.058 | 0.4686 |  | **7.96E-09** |
| CTC8 | 8.417 | 8.564 | 10.87 | 0.9249 | 10.68 | 10.28 | 9.806 |  |
