# Supplementary material for: Shepherding the past: High-resolution data on Neolithic Southern Iberian livestock management at Cueva de El Toro (Antequera, Málaga)
Source: PLoS One. 2024 Apr 3;19(4):e0299786. doi: 10.1371/journal.pone.0299786 (PMC10990244; doi:10.1371/journal.pone.0299786)
Supplement: S5 Table — Results from the calculation of the best fit for combined variation of X (period), A (amplitude), x0 (delay), M (mean) and Pearson correlation. (DOCX) [file pone.0299786.s005.docx]

**S5 Table. Modelling of δ^18^O sequences.** Results from the calculation of the best fit for combined variation of X (period), A (amplitude), x0 (delay), M (mean) and Pearson correlation.

| **Sample** | **CTC1 M3** | **CTC19 M3** | **CTC5 M3** | **CTC7_M3** | **CTC8_M3** |
| --- | --- | --- | --- | --- | --- |
| X | 23.98 | 28.60 | 32.68 | 21.43 | 25.39 |
| A | 1.02 | 0.88 | 0.84 | 1.23 | 1.47 |
| x0 | 9.64 | 24.53 | 31.33 | 17.24 | 4.81 |
| M | 0.38 | -0.52 | 0.82 | -0.19 | -0.45 |
| Pearson | 0.93 | 0.98 | 0.92 | 0.95 | 0.94 |
| x0/X | 0.40 | 0.86 | 0.96 | 0.80 | 0.19 |
