# Supplementary material for: Shepherding the past: High-resolution data on Neolithic Southern Iberian livestock management at Cueva de El Toro (Antequera, Málaga)
Source: PLoS One. 2024 Apr 3;19(4):e0299786. doi: 10.1371/journal.pone.0299786 (PMC10990244; doi:10.1371/journal.pone.0299786)
Supplement: S1 Code — (DOCX) [file pone.0299786.s006.docx]

**S1 Code. Bayesian model code for the radiocarbon dates of the Cueva de El Toro**

Plot();

{

Sequence()

{

Boundary("Start");

Phase("layer")

{

R_Date("CIRAM-5475", 6230, 35);

R_Date("CIRAM-5476", 6232, 35);

R_Date("CIRAM-5477 ", 6172, 35);

R_Date("CIRAM-5478", 6113, 36);

R_Date("CIRAM-5479", 6158, 33);

R_Date("CIRAM-5480", 6242, 33);

R_Date("CIRAM-54781", 6201, 33);

R_Date("CIRAM-5482", 6185, 33);

Interval("Duration");

};

Boundary("End");

};

};
