# Supplementary material for: Shepherding the past: High-resolution data on Neolithic Southern Iberian livestock management at Cueva de El Toro (Antequera, Málaga)
Source: PLoS One. 2024 Apr 3;19(4):e0299786. doi: 10.1371/journal.pone.0299786 (PMC10990244; doi:10.1371/journal.pone.0299786)
Supplement: S1 File — (DOCX) [file pone.0299786.s007.docx]

**S1 File. Protocol for carrying out radiocarbon analysis and results of each sample with the quality control data**

The samples were sent to the CIRAM laboratory, where they were processed and analysed according to the following protocol:

Samples containing collagen are treated with hydrochloric acid (1 M) at cold temperatures for 24 hours to eliminate surface contaminations and partially degrade the mineral portion of the bone, enhancing collagen extraction efficiency. Those containing cellulose undergo treatment with hydrochloric acid (1 M) at 80 °C for 1 hour to remove surface contamination, followed by treatment with sodium hydroxide (0.1 M) at room temperature for 10 minutes to eliminate residual humic and fulvic acids. After a second hydrochloric acid treatment to prevent atmospheric CO2 absorption from the previous basic treatment, collagen-containing samples are gelatinized.

Each sample undergoes combustion at 920 °C, transforming into gas. During this stage, the C/N ratio is initially checked using an elemental analyzer (Elementar Vario ISOTOPE Select). Residual carbon dioxide (CO2) is separated from other combustion residues using a zeolite trap. The residual CO2 is converted into graphite using an automated system (AGE 3, Ion Plus) through catalysis, following the method described by Vogel et al. (1984, Nuclear Instruments and Methods in Physics Research Section B: Beam Interactions with Materials and Atoms, 5 (2), 289-293).

Carbon isotopes are separated by mass spectrometry using a 250 kV accelerator in collaboration with JSC Barnas (ISO 9001 and ISO 14001). The 14C concentration is determined by simultaneously comparing measurements of 14C, 13C, and 12C with those in reference products (oxalic acid, CO2 standard, coal). The δ13C and δ15N ratios are separately measured on a stable isotope ratio mass spectrometer with an error below 0.1‰ (IRMS, Elementar Isoprime precisION). Measured isotopic compositions are normalized using a calibration curve constructed from the measurement of the following reference standards: IAEA 600 caffeine (δ13C = -27.771±0.043‰ VPDB, δ15N = +1.0±0.02‰ Air), BCR 657 glucose (δ13C = -10.76±0.04‰ VPDB, European Commission certificate EUR 20064 EN), and IAEA N 2 ammonium sulfate (δ15N = +20.41±0.12‰ Air).

The conventional 14C age was calculated using the method described by Stuiver and Polach (Radiocarbon, 19 (3), 1977, 355 363). It takes into account the isotopic fractionation correction (δ13C), based on a comparison of the 13C/12C and 14C/12C concentration ratios. This factor is used to control the effects of any pollution and to assess the reliability of the measurement: it is a good indicator of the 'quality' of the sample. The measurement uncertainty associated with the result combines the statistical uncertainties of counting the residual 14C, the variability of the measurements and the effects of subtracting the "blank".

| **Lab code** | **Sample** | **Nature** | **C/N** | **pMC corrigé** | **d13C (‰)** | **d15N (‰)** |
| --- | --- | --- | --- | --- | --- | --- |
| CIRAM-5475 | S1 | Bone (mandible) | 3.26 | 46.04 ± 0.2 | -19.48 | 4.78 |
| CIRAM-5476 | S2 | Bone (mandible) | 3.24 | 46.03 ± 0.2 | -19.55 | 6.43 |
| CIRAM-5477 | S4 | Tooth root | 3.23 | 46.38 ± 0.2 | -20.2 | 7.23 |
| CIRAM-5478 | S5 | Tooth (Dentine) | 3.25 | 46.72 ± 0.21 | -18.02 | 7.49 |
| CIRAM-5479 | S7 | Tooth root | 3.29 | 46.46 ± 0.19 | -20.15 | 7.51 |
| CIRAM-5480 | S8 | Tooth root | 3.22 | 45.98 ± 0.19 | -15.97 | 6.92 |
| CIRAM-5481 | S19 | Bone (mandible) | 3.23 | 46.21 ± 0.19 | -20.39 | 5.04 |
| CIRAM-5482 | S21 | Bone (mandible) | 3.25 | 46.3 ± 0.19 | -19.41 | 6.27 |
